# From civilian service to military service: what led policy-makers to remove nursing care from field units of the Israeli defense force (IDF) and return it later?

**DOI:** 10.1186/s13584-019-0360-2

**Published:** 2019-12-31

**Authors:** Ronen Segev

**Affiliations:** Department of Nursing, Ruppin Academic Center, 4025000 Emek-Hefer, Israel

**Keywords:** Military nursing, Israel defense forces, Military hospitals, Civilian and military nursing

## Abstract

**Background:**

From the very onset, Israeli military nurses served in supporting positions on the front lines, shoulder to shoulder with men. When the IDF was established in 1948, nurses were sent to serve near areas of conflict and were not included in compulsory military service in field units. Once the military hospitals were closed in 1949, nursing in the Medical Corps lost a clear military purpose, and its main contribution was in the civilian arena. From 1949 until 2000, most recruited military nurses operated their mandatory service mainly in a civilian framework according to the integration agreement between the ministry of defense to the ministry of health. Between 2000 to 2018, military nurses served at home front military clinics and in headquarters jobs at the Medicine Corps. In2018, the Medical Corps decided to integrate military nurses into the Israeli military service in order to cope with the shortage of military physicians, among other things, and ensure appropriate availability of medical and health services for military units.. This study examines, for the first time, the considerations that led to the closure of military hospitals and the transfer of the military service of nurses in the IDF to the Ministry of Health in 1949 and the decision in 2018 to return the military nurses to the field’s military battalions.

**Methods:**

The study was based on an analysis of documents from the IDF archives, the Israeli parliament archive, the David Ben-Gurion archive, articles from periodical newspapers, and interviews with nurses and partners in the Israeli Medical Corps.

**Results:**

During almost 70 years, Israeli military nursing’s main contribution was to the civilian hospitals. The return of nursing care to the IDF field units in recent years intended to supplement the medicine corps demands in field units by placing qualified academic nurses.

**Conclusions:**

The removal of nursing care from the IDF field units was provided as a response to the needs of the health demands of the emerging state. Until 2018 there was no significant need for military nurses except in emergency time. This is in contrast to other military nursing units.

## Background

On 2 February 1901, an independent American Army Nurse Corps was established in order to organize the recruitment of trained nurses during wartime [1]. Initially, army nurses were volunteers, and though many served in the major military hospitals on American soil, the vast majority served abroad [2]. However, with the entry of the United States into WWII, the Cadet Nurse Corps was founded to cope with the nursing shortage, and was signed into law by President F.D. Roosevelt on 1 July 1943. As a result, more than 54,291 American nurses were drafted to serve in the war [2]. Britain developed in parallel the Queen Alexandra’s Royal Army Nursing Corps (QARANC). Officially founded in 1902, the British nursing service was established on the foundations of the earlier Army Nursing Service established in 1881, which can trace its origins to Florence Nightingale [3]. In Canada, the Army Medical Corps was established in 1904, and included a permanent nursing force. By the first world war, thousands of nurses with civilian qualifications were drafted overseas to serve in military hospital wards, tents, ships, and trains. Those nurses who were always women,received equal payment and had equal ranks to men [4].

Military nurses in all three countries underwent training in addition to their medical training to serve as commanders in military hospitals and cope with military tasks, such as caring for wounded soldiers on sea, air, and land [5]. During World Wars I and II, thousands of American, British, and Canadian army nurses aided wounded soldiers in war zones around the globe [6]. Military nursing was officially founded in these three countries and organized as a professional nursing force to heal and nurse the wounded soldiers of each country overseas.

During the early twentieth century, Israeli military nurses were found mostly in the home front, dealing with both civilian and military issues, without any protocols or guidelines. Following United Nations (UN) resolution 181 on 29 November 1947-the establishment of the state of Israel, the nascent country of Israel was flung into a full, outright war for its very existence: the 1948 Arab-Israeli War. The war ended on 20 July 1949, followed by a period of relative quiet, but Israel had a massive influx of new immigrants that included the survivors of the Holocaust and substantial numbers of Jews from Arab countries (e.g., Morocco, Iran, Yemen, Iraq), all of whom came with their own medical problems.

### Fighting forces and medical care

Military nurses were active even before the establishment of the state of Israel in 1948 and volunteered to protect the Jewish communities in British Palestine. When the IDF was established in 1948, nurses were sent to serve near areas of conflict. This unique policy was a result of the IDF’s high command policy and the proximity of the front to the civilian rear in the armistice border .

Military nursing in Israel is unique in that it has leaned towards the civilian health sector from its inception, which is attributed to the massive influx of immigrants after the Arab-Israeli War. Another unique characteristic of the IDF nursing service is related to the organization of the military medical services in Israel, which were established alongside, and even as a part of, civilian hospitals. This is in contrast to what is customary in other militaries, such as in the United States and the United Kingdom, where military training and care services are completely separate from civilian services [7].

The Arab-Israeli War was long and grueling, and the hastily organized, highly undertrained, and minimally supplied military medical personnel realized the need for a medical service that was more advanced and able to provide medical care at any time and place, even at the remotest border settlements. At that point, wounded were transported to civilian hospitals for hospitalization, and their care was managed in an unorganized manner. What were needed were medical units (doctors, nurses, and other medical assistants) that could accompany the fighting forces and quickly evacuate the wounded to military hospitals [8].

In February 1948, Dr. Haim Sheba was appointed as the first head of the IDF Medical Corps. He was given the task of establishing and administering the IDF’s medical services. As a first step in initiating the Military Medical Services, Sheba began widespread recruitment of physicians under 45 years of age and unmarried nurses [9, 10]. By the end of 1948 the Military Medical Services included 5814 medical staff members [11]. In addition, Dr. Haim Sheba founded 16 military hospitals throughout the country. The largest were the military hospitals set up at Tel HaShomer (today Sheba University Medical Center) and Tzrifin-Assaf Harofeh (today the Shamir medical center), which comprised 3500 hospitalization beds at their peak, almost 3-times the number of beds in the civilian sector (1166). In 1948, a total of 263 nurses were serving in military hospitals [12].

Toward the end of 1949, the Military Medical Service became the Medical Corps, which operated as an independent body within the IDF. The Medical Corps provided an organized operational perception that was integrated into the IDF’s activity in the front and rear lines. After the 1948 Arab-Israeli War, the Corps’ structure was narrowed, with its main activity being relegated to preventive care in the units and rehabilitation of the war’s wounded and disabled [11].

### Conscription of women and nursing professionals into the IDF

From its inception on 26 May 1948, the conscription policy of the IDF included both genders, with all able-bodied men and women required to serve. This was a combination of the egalitarian nature of the early pioneers, which was echoed in the official policies of the new state, and the practical consideration that all available resources needed to be harnessed for the war effort [13]. The policy was 3 years for men and 2 years for women, followed by reserve duty. Initially, the policy applied to all women, both single and married (but without children) [14].

As nursing was a very common profession of women, it was natural that nurses, nursing assistants, and other hospital professionals constituted a significant proportion of female conscripts. Also, as they were considered vital professionals, they were generally recruited to the Medical Corps and not the Women’s Corps [15]. Some early conscripts were even appointed to key positions and helped establish the IDF’s Medical and Nursing Unit [16–18].

However, the civilian sector also needed nursing personnel, and there was ongoing competition between these two sectors, including competition for nurses from abroad who rallied to support the fledgling state [19]. In the archival files, no systematic and accurate registration is found regarding the number of nurses recruited to the military annually, but partial data suggest that only 10–15% of the approximately 150–200 nurses who graduated civilian nursing school,which most of them located within or next to general hospitals, annually were recruited [20]. Thus, the recruitment and organization of the nurses was difficult to achieve during the first decade of the IDF.

### The “medical war” on the civilian front and changing the designation of nurses in the IDF

After the Arab-Israeli War, when a significant portion of the wounded had been rehabilitated and released home, the resources were directed tothe civilian front. There was a pressing need to address the medical deficiencies in the civilian medical field, which was under increasing stress due to the massive Jewish immigration that began after the State was secured.

The first step was to transfer budget; the General Staff was ordered to reduce the scope of the Medical Corps by reducing the total number of medical personnel and decrease expenses in the military hospitals. Second, military hospital beds were reassigned to the Ministry of Health, contingent upon two conditions: 1) in times of war and according to the Medical Corps’ demand, the hospitals would be returned to the IDF’s authority, and 2) that the care and rehabilitation of disabled soldiers be taken over by the Ministry of Health [21]. By March 1949, 1000–1200 hospital beds had been transferred from the military for civilian use [22]. Some medical personnel were also reassigned [23]. Third, as the two largest military hospitals, Tel-Hashomer and Tzrifin, were geographically close to one another and neither was at full capacity, Tzrifin was transferred to the Ministry of Health. Two other military hospitals, in Haifa and Nes Ziona (near Tel-Aviv), were also transferred to the Ministry of Health. The Military Hospital in Jerusalem was transferred to the Hadassah Medical Organization.

It was decided that Tel HaShomer would become the military’s main medical facility and used as a continuing education center for medical staff; in times of emergency, it would be adjusted to accommodate the hospitalization needs of the IDF. However, in 1953, during the austerity following the financial crisis, Tel HaShomer was also transferred to the Ministry of Health [24]. Not long after, in 1955–1956, as a result of rising tensions along the borders because of Fedayeen Death Squad attacks, a governmental committee discussed the need to secure the allocation of civilian hospitalization beds to the IDF in times of emergency [25]. Thus, on 5 February 1957, the Ministry of Defense and the Ministry of Health signed an agreement known as the Integration Agreement, by which the Ministry of Health agreed to give full medical and ambulatory (without hospitalization) care to IDF soldiers in times of tranquility and emergency care at the four hospitals under its authority, including any medical procedures and medications necessary. In exchange for this bundle of medical services, the Ministry of Defense allocated military nurses to serve in the civilian hospitals under the authority of the hospital’s director, without compromising any of their rights and duties associated with their formal status as soldiers [26].

### IDF nurses’ service at integrated hospitals

Between 1949 and 1956, when military hospitals were under the jurisdiction of the civilian sector, the majority of military nurses worked in civilian hospitals as part of their military service. Though the contributions of the military nurses were important in the civilian hospitals in the center of the country, they were particularly important at hospitals operating in peripheral areas of the country, where few, if any, registered nurses lived. For example, in 1959, upon graduating from her studies at the Tel HaShomer nursing school, Nurse Aliza Toledano was posted to Poriyah Hospital (near Tiberias) in northern Israel. Three years later, she was promoted to head nurse. According to an interview with her, within 1 year of service, the military nurses significantly improved the level of performance at the hospital [27]. The majority of recruited nurses were sent to “integrated hospitals”. A few of them, roughly 14 nurses per year, were sent to serve in patient rooms on military bases, and only one was assigned to instruct at the military medical school each year [28, 29].

Although the concept behind the idea of integrated hospitals was justified theoretically, the policy of allocating nurses as an essential medical human resource from the military authority to the civilian ward had a major influence on the future of Israeli military nursing. The policy essentially meant that the function of the civilian health system depended on military nurses. This hindered the nurses’ professional development and their ability to attain significant military positions in the Medical Corps. Furthermore, the majority of recruited nurses found themselves serving in a civilian arena with day-to-day routine demands; they did not have any experience with nursing demands in emergencies. Therefore, ironically, during emergencies, the majority of nurses recruited to field hospitals during the Israeli wars between 1948 and 2000 were civilian, based on their professional and management experience in the operating room, intensive care units, and emergency department. This met the military hospital’s needs, despite the nurses’ lack of military background and training. Working in the military milieu was sometimes a totally new experience for them, and they were forced to assimilate into the military setting quickly and under pressure of the circumstances (i.e., war or other emergency). One of their major contributions in the emergency and disaster arenas is attributed to the nursing care and management function in global humanitarian missions [7, 30].

However, the hands of both the head of the military nurses and the head of the Medical Corps were tied due to the integrated hospitals agreement, which remained valid and enforced in the military nurses’ service until 2000. Although lacking documentation and archive evidence of the number of nurses recruited over the years, one of the reasons for the termination of the agreement may lie in the decline in the number of nurses recruited to the IDF due to the transformation of nursing training programs to academic programs [31].

During almost 70 years, Israeli military nurses served in the civil sector, far from the significant military field territory.

Between 2000 and 2018, after the termination of the integration agreement, military nurses served at home front military clinics and in headquarters jobs at the Medicine Corps.

### The return of IDF nurses to military field units

Until the mid-1980s, the roles of women in military service in the IDF was perceived as insignificant in Israeli society. Since then, an increase in feminist awareness led to the opening of male positions for women in the IDF (i.e., pilots, naval commanders, border police). However, military organizations were resistant to a gender change, and the number of positions opened to women in the IDF and their integration in field units was slow and limited [32, 33]. This claim may explain the slow change in the Medical Corps attitude toward nurses’ deployment in new positions in general and in field units in particular [7]. In addition to the military-gender change, in recent decades, policymakers in many countries throughout the world, including Israel, have expressed concerns about the severe shortage of physicians for economic, social, and demographic reasons, with wages, workplaces, and the number of hours worked each week as crucial factors in choosing medical studies [34]. The general shortage of physicians directly affects the army as well. The severe gap in medical personnel required for the military environment forced the heads of the Medical Corps to find a creative solution for the education and training of military physicians [35]. Parallel to the shortage of physicians, in 2009, the Ministry of Health began to expand nurse authorities in Israel under the influence of a global nursing professionalism process. As a result, nurses were allowed to prescribe medication as part of a new training program in clinical specialties and qualified as a nurse practitioner in the health care system. This process did not skip the military nurses. Upon enlisting in the IDF, they began commanding and professional training courses, such as primary care nursing. In this course, the nurses learn the principles of treating soldiers in the primary medicine clinic at the training bases in combat units. In this way, the burden on physicians is reduced and the availability of care for soldiers is improved [36]. The integration of academic nurses into field units alongside physicians was one of the solutions reached for the shortage of military physicians. Therefore, the Medical Corps plans to increase the number of students studying nursing each year from 25 to 120. These students are studying for 3 years instead of 4 in a training program tailored for the army. Accordingly, in 2018, four male nurses and one female nurse were assigned to combat units for the first time as a pilot program and to provide advanced medical treatment for soldiers [37]. This step promotes the professional prestige of military nurses while saving economic resources for the army, as the cost of training a physician for 7 years is significantly higher than the period of training for nurses, which lasts only 3 years.

## Conclusion

Almost from its very inception, and except for times of emergency and war when medical personnel were needed on the frontlines, the service route of the military nurse in Israel has leaned towards the civilian health sector. A unique characteristic of the IDF’s nursing service is the organization method of the military medical services in Israel, which were established alongside civilian hospitals and not as separate training and care services. These are in direct contrast to several other militaries, including the United States and United Kingdom, where military medical personnel serve only the military population.

**Fig. 1:**
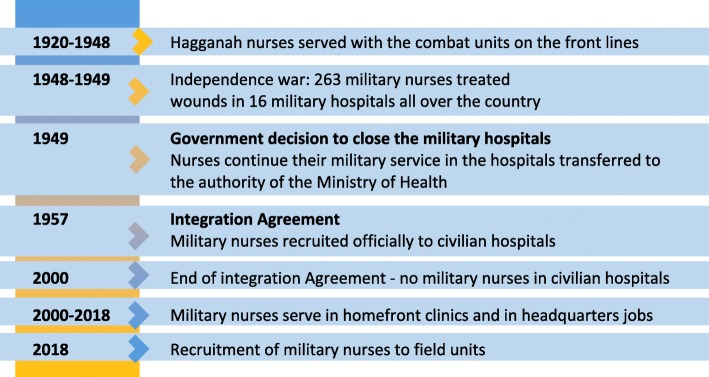
Military nurses in Israel

The closing of the military hospitals in Israel in 1950s was a turning point for nurses’ professional future in the IDF. From then on, most recruited military nurses operated on behalf of the military (i.e., fulfilling their mandatory service), but served mainly in a civilian framework (Fig. 1). On the other hand, at times of war, most of the nurses who served in field hospitals near the front were civilian nurses with no military experience. This was a result of the lack of professional human resources. Throughout, there was a certain tension between the military and civilian sectors over “possession” of medical human resources.

Military nurses fulfilled a multitude of social and national tasks, such as caring for the wounded, caring for immigrants in transit camps, and helping at the integrated peripheral hospitals, and their dedication and often selfless devotion is a source of admiration despite the hardships and changing conditions military nurses have had to endure.

However, as a military institution, at the end of the IDF and the State of Israel’s first decade, Israeli military nursing was still not fully organized, there was no special training for military nurses, and there were no organized plans for recruitment and professional functioning during wars and time of conflict. Until 2018 there was no significant need for military nurses except in emergency time. It has been changed in 2018 when military nurses filled the gap created by the physician shortage and, in response to the gender-changing process in the army and the global nursing professionalism trend, their contribution to combat field units was highly anticipated.

## Data Availability

The datasets used and analysed during the current study are available from the corresponding author on reasonable request.

## Abbreviations

IDF: Israeli Defense force
QARANC: Queen Alexandra’s Royal Army Nursing Corps
UN: United Nations
